# Association of intracellular lipid accumulation in subcutaneous adipocyte precursors and plasma adipokines in bariatric surgery candidates

**DOI:** 10.1186/s12944-019-1081-9

**Published:** 2019-06-13

**Authors:** Ioana Hristov, Veronica Mocanu, Florin Zugun-Eloae, Luminita Labusca, Iustina Cretu-Silivestru, Teodor Oboroceanu, Crina Tiron, Adrian Tiron, Alexandrina Burlacu, Alin Constantin Pinzariu, Ioana Armasu, Radu Mircea Neagoe, Adrian Covic, Viorel Scripcariu, Daniel Vasile Timofte

**Affiliations:** 1 “Grigore T. Popa” University of Medicine and Pharmacy, 16 Universitatii Str., 700115 Iasi, Romania; 2 TRANSCEND Research Center, Regional Institute of Oncology, Iasi, Romania; 3 Stem Cell Laboratory, National Institute of Research and Development for Technical Physics (NIRDTP), Iasi, Romania; 4 ”Nicolae Simionescu” Institute of Cellular Biology and Pathology of the Romanian Academy, Bucharest, Romania; 5 University of Medicine, Pharmacy, Sciences and Technology, Tg Mures, Romania; 6 Academy of Medical Sciences, Bucharest, Romania

**Keywords:** Adipocyte differentiation, Subcutaneous adipose derived stem cells, Obesity, Metabolic syndrome, Insulin resistance, Leptin/adiponectin ratio, Metabolic surgery

## Abstract

**Background:**

The adipocyte expansion is a critical process with implications in the pathogenesis of obesity associated metabolic syndrome. Impaired adipogenesis leads to dysfunctional, hypertrophic adipocytes, local inflammation and peripheric insulin resistance.

**Methods:**

We assessed the relationship between the adipogenic differentiation capacity of the subcutaneous adipose derived stem cells (ASCs), evaluated by total lipid accumulation, and the metabolic and hormonal profile in a group of obese female patients proposed for bariatric surgery (*N* = 20) versus normal weight female controls (*N* = 7).

**Results:**

The lipid accumulation (measured as optical density at 492 nm) of ASCs during their differentiation to adipocytes was significantly lower in ASCs isolated from obese patients as compared to ASCs isolated from normal weight patients (0.49 ± 0.1 vs. 0.71 ± 0.1, *p* < 0.001). Significant negative correlations between lipid accumulation in adipogenic differentiated ASCs and plasma concentrations of triglycerides (*p* < 0.01), insulin (*p* < 0.001), HOMA-IR (p < 0.01), adiponectin (*p* < 0.05) and leptin/adiponectin ratio (*p* < 0.05) were found in obese group.

**Conclusions:**

In severely obese female patients, the abnormal adipogenesis is related to insulin resistance and leptin/adiponectin ratio. The abnormal lipid accumulation in the mature adipocyte derived from obese ASCs could possible predict the further development of type 2 diabetes mellitus in severely obese patients and influence the selection of patients for bariatric surgery.

## Background

The obese individuals are reportedly at high risk for cardio-metabolic diseases. As the metabolic syndrome is an important predictor, not only for cardiovascular mortality but also for all-cause mortality, the evaluation of the metabolic status of the obese patients needs to be performed precociously and by accurate methods [1].

The current definition of the International Diabetes Federation (IDF) for the metabolic syndrome [2] includes the assets of the cardiovascular risk factors that are considered the best predictors for cardiovascular mortality for obese and non-obese patients and includes parameters that can be accessible for screening [3]. Insulin resistance evaluation by HOMA-IR is considered as a good cardiovascular risk predictor [4], being also demonstrated as a valuable criteria for recognition of the obese individuals with a higher mortality risk [1].

However, around 35% of the obese individuals do not develop insulin-resistance or associated metabolic disturbances [5], and the particularities of adiposity expansion in the obese patients in not understood.

Insulin resistance is closely associated with disturbances of fat metabolism [6]. Thus, exceeding the storage capacity of the subcutaneous adipose tissues results in lipotoxicity, a condition characterized by fatty acid infiltration of insulin target tissues i.e., skeletal muscle and liver, that eventually leads to insulin resistance [6].

High leptin levels and leptin:adiponectin ratio are predictors of obesity related complications as type 2 diabetes mellitus (T2DM) and hypertension independent of BMI or the metabolic syndrome (MetS) criteria [7–9].

The subcutaneous fat mass expansion is initiated by adipocyte hyperplasia, a physiological process that generates new mature adipose cells through activation and differentiation of multipotent stem progenitors. However, increasing expansion requirements eventually exceeds the individual adipogenic differentiation capacity of preadipocytes. When this adipogenic potential is reached, excessive lipid accumulation results in a dysfunctional adipose tissue [10–14], due to adipocyte hypertrophy, decreased adipogenesis and angiogenesis [15, 16].

Subcutaneous adipose tissue consists predominantly of adipocytes, but also contains other cell populations generally referred to as the stromal vascular fraction (SVF). Zuk et al. [17] identified in the SVF, a multipotent, undifferentiated, self-renewing progenitor cell population that is morphologically and phenotypically like mesenchymal stem cells (MSCs). These isolated adipose tissue-derived stem cells (ASCs) display a capacity of differentiation like MSCs and show the expression of the specific stem cell markers in vivo [18]. ASCs however have a series of advantages as a multipotent differentiation source as they are more accessible by simple subcutaneous adipose tissue biopsy, a repeatable minimally invasive method, the isolation procedure is simple and the stem cell quality and proliferation capacity that does not decline with the age of the patient [19]. After isolation and proliferation of these ASCs, they can be used for experimental study of the molecular processes in regulating adipocyte differentiation [20].

Previous studies evaluated the changes induced by obesity in the adipogenic differentiation of ASCs with controversial results: from enhanced [21, 22], unchanged [23] to decreased adipogenesis [24–27]. This could be attributed to ASCs isolated from obesity donors with distinct stages of obesity, different ages, as well as with or without metabolic dysfunction.

The aim of our experimental study was to assess the adipogenic capacity of subcutaneous ASCs in obese female patients prior to bariatric surgery in relation to plasma metabolic and hormonal parameters.

## Materials and methods

### Characteristics of the study group

The study included 20 obese female patients (OB group), referred for Laparoscopic Sleeve Gastrectomy (LSG) and seven normal weight females (NW group, control) with other abdominal surgery indications. The mean body mass index (BMI) was 45.02 ± 6.31 kg/m^2^ in OB group and 24.46 ± 2.50 kg/m^2^ in NW group. The age ranges of the two groups matched, with a mean of 42.05 ± 9.91 years for OB group and 42.00 ± 10.67 years for the NW group.

### Biochemical measurements

Blood samples were collected after 12 h of fast, before the bariatric surgery procedure. The lipid profile was established based on the following serum determinations: Fasting plasma glucose (FPG), Total Cholesterol (TC), Low Density Lipoproteins (LDL) Cholesterol, High Density Lipoproteins (HDL) Cholesterol, Triglycerides (TG) and TC/HDL ratio. Plasma concentrations of insulin and total morning cortisol (8:00 a.m) were measured using electrochemiluminescence immunoassay method. HOMA-IR was calculated using the formula: $\frac{FPI\ x\  FPG}{405}$, where FPI = fasting plasma insulin (μUI/ml) and FPG = fasting plasma glucose (mg/dl). Leptin and adiponectin was measured in plasma using ELISA method (Sigma Aldrich kit RAB0005 was used for adiponectin and Fine Test EH0216 for leptin serum concentration quantification).

### ASCs isolation and proliferation

ASC culture was derived using a protocol described by Lyons [26], starting from a small amount of subcutaneous abdominal adipose tissue (less than 1 g) remained in the trocar during the laparoscopic procedure, so that to not create supplemental patient discomfort to the bariatric surgery procedure. The sample was washed in 0.9% saline solution and digested in 10 mg/ml collagenase type I (Sigma Aldrich lot # SLBM2283V) for 15 min. Dulbecco’s modified Eagle’s medium (DMEM)/F12. The washed digested tissue was subjected to red blood cells lysis and released cells were passed through a 70 μm-cell strainer before being re-suspended at a density of 1.5 × 10^5^ cells/ml culture medium (DMEM/F12 + 10% foetal bovine serum (FBS),100 U/ml penicillin and 100 μg/ml streptomycine). Cells were cultured at 37 °C under 5% CO_2_ atmosphere and the medium was refreshed every 2–3 days. Progression towards ASCs confluence was followed for a medium duration of 16 ± 3 days using an inverted optical microscope (Olympus CKXC3).

### Adipogenic differentiation

The 80–90% confluent ASCs are incubated in an adipogenic induction medium containing: DMEM/F12, 10%FBS, 0.5 mM IBMX, 1% ITS (Insulin Transferrin Selenite, Sigma Aldrich)., 200 μM Indomethacin (Sigma Aldrich), 1 μM Dexamethasone, The induction medium was changed every 2–3 days, with an alternative use of induction and maintenance medium (DMEM/F12 + 10% FBS + 1% ITS), until the day 21, when a complete adipogenic differentiation was observed. Undifferentiated control cells were run in parallel with cells cultured in maintenance medium only.

### Oil red O staining

To determine the lipid accumulation, cells were fixed in 10% formalin for 20 min and stained with 0.1% Oil Red O (ORO) for 60 min as described by Kraus A et al. (2016).

Lipids were spectrophotometrically quantified using a microplate reader Tecan Sunriseby reading the absorbance values at 492 nm wavelength (OD_492_), after elution of ORO-stained cells in 90% isopropanol. Absorbance values for each plate expressed the lipid accumulation quantification.

### Immunofluorescence analysis

To evaluate the mesenchymal features of the cells, a monoclonal anti-vimentin antibody (Cell Signaling Tech, D21H3) coupled with Alexa Fluor® 488 was used for immunofluorescent labelling of control and differentiated cells. To these aims, cells were permeabilized in Triton X100 (0.1% in PBS) and incubated with the antibodies, as previously described [27]. To identify the adipocyte specific markers, immunofluorescence staining for peroxisome proliferator activated receptor gamma, PPAR γ (Cell Signaling Tech, 2435S) and perilipin (Thermo Fisher Scientific, PA5–18694) will be performed on differentiated ASCs. At the end of the procedures, the stained cells were washed and evaluated under a fluorescent microscope ZeissAxio Observer Z1 microscope. DAPI (Sigma, D9542) was used to stain cell nuclei.

### Statistical analysis

Data were analysed using IBM SPSS Statistics 21 Software and expressed as mean (±standard deviation) and median (min-max) values. Comparisons between parameters in obese patients and normoponderal controls were performed using the Mann–Whitney test. Kendall’s Tau-b correlation coefficient, a nonparametric measure, was computed to evaluate correlations between lipid accumulation evaluation and biochemical and hormonal parameters. Significance was defined as *p* < 0.05.

## Results and discussion

### Characteristics of patients

Plasma concentrations of total cholesterol, LDL-cholesterol, triglycerides, fasting glucose, insulin, C-peptide and metabolic indices (HOMA-IR and total cholesterol/HDL-cholesterol) were significantly increased in OB group as compared with NW group. Significantly higher plasma concentrations of leptin and leptin:adiponectin ratio values were found in OB group as compared with NW group. No significant diferences were obtained for plasma morning cortisol and adiponectin (Table 1).

**Table 1:** Characteristics of the obese patients and normal weight controls. The data are presented as the mean (± standard deviations) and median (min-max) values

Parameter	Normal weight controls (*N* = 7)	Obese patients (*N* = 20)	*P*- value
Age (years)	42.0 (10.7)/42.0 (25–58))	42.1 (9.9)/45.5 (23–63)	0.978
BMI (kg/m^2^)	24.5 (2.5)/24.7 (21.2–29.0)	45.0 (6.3)/43.7 (36.4–58.9)	< 0.001
Lipid profile:			
Total cholesterol (TC)(mg/dl)	166.7 (18.3)/159.0 (151–202)	210.6 (32.1)/212.5 (138–265)	0.006
LDL-cholesterol (LDL)(mg/dl)	90.0 (2.8)/90.0 (86–93)	141.0 (29.0)/139.5 (80–184)	0.001
HDL-cholesterol (HDL)(mg/dl)	54.2 (3.7)/54.0 (48–59)	49.7 (12.2)/49.0 (23–75)	0.223
TC/HDL (Normal< 4)	3.1 (0.3)/3.1 (2.7–3.4)	4.4 (1.0)/(3.1–6.2)	0.001
Triglycerides (TG) (mg/dl)	96.3 (14.4)/93.0 (73–115)	165.3 (103.5)/140.5 (69–448)	0.025
Glucose homeostasis:			
Fasting plasma glucose (mg/dl)	86.9 (8.9)/87.0 (73–99)	126.8 (74.1)/102.0 (77–362)	0.012
Insulin (μIU/ml)	7.4 (1.6)/6.89 (6.3–10.7)	16.8 (9.1)/15.8 (6.0–43.4)	0.003
C-peptide (ng/ml)	1.6 (0.3)/1.5 (1.2–2.1)	3.2 (1.0)/3.5 (1.2–4.9)	0.013
HOMA-IR	1.6 (0.4)/1.5 (1.3–2.5)	6.1 (7.0)/3.8 (1.4–28.7)	0.001
Plasma hormones			
Morning Cortisol (μg/dl)	10.1 (3.7)/9.6 (6.2–15.6)	12.9 (4.1)/12.4 (7.6–23.4)	0.072
Leptin (ng/dl)	5.7 (2.0)/6.0 (2.8–8.0)	26.5 (8.8)/27.6 (8.0–39.6)	< 0.001
Adiponectin (μg/dl)	30.6 (2.0)/30.9 (2.7–3.4)	29.0 (3.2)/29.1 (1.9–3.3)	0.166
Leptin: Adiponectin Ratio	0.18 (0.7)/1.9 (0.9–2.7)	0.93 (0.3)/1.0 (2.6–14.4)	< 0.001
Adipocyte differentiation dysfunction			
Lipid accumulation (OD492)	0.71 (0.1)/0.75 (0.50–0.82)	0.49 (0.6)/0.48 (0.41–0.67)	< 0.001

*P*-values were assessed by Mann–Whitney test to compare non-normal distributed variables

*P*-value ≤ 0.05 was considered significant

### Adipose derived stem cell (ASCs) isolation, proliferation and differentiation

The stromal fractions were obtained from subcutaneous abdominal adipose tissue derived from OB and NW group and grown in culture at a starting density of 1.5 × 10^5^ cells/ml.

The ASCs proliferation to confluence was obtained after 18 ± 4 days for OB patients isolated ASCs and after 16 ± 3 days for ASCs in NW patients, with no statistically significant difference between OB and NW groups.

The cell culture viability was evaluated by MTT-formazan (3, 4, 5-dimethylthiazol-2,5-diphenyltetrazolium bromide) according to the protocol of Mossman et al. [28]. The metabolic activity of mesenchymal stem cells evaluated using MTT assay was 93 ± 3% for the OB group with a similar result: 94 ± 2% for the control NW group.

### Identification of mesenchimal specific markers

**Fig. 1:**
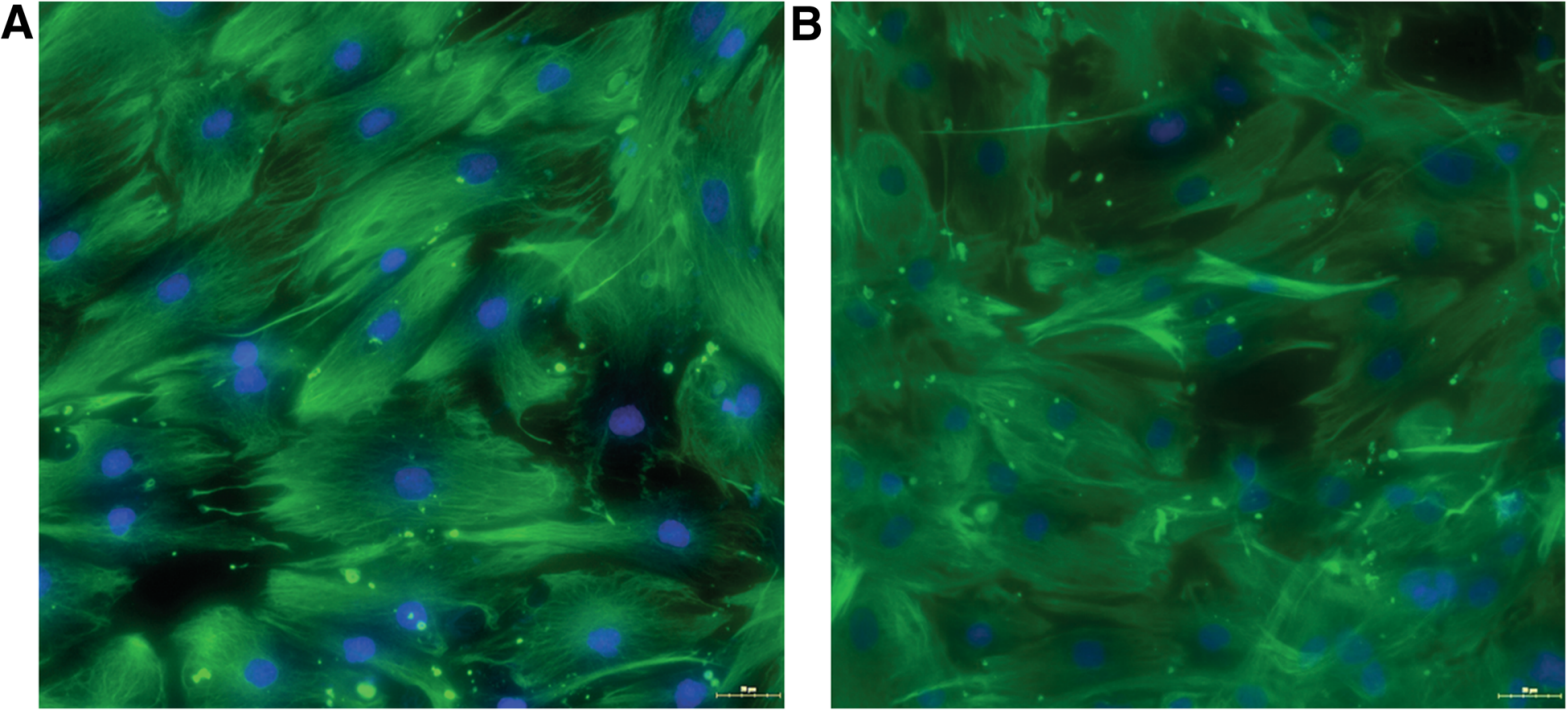
ASCs characterisation as mesechymal cells using Vimentin IF stain. Merge image cytosolic Vimentin (green) and nuclear DAPI (blue) **a**. Normoponderal ASCs **b**. Obese ASCs

In our study, in order to characterize thecell culture population as mesenchymal stem cells, we evaluated the expression of vimentin (Fig. 1).

### Differentiation of subcutaneous ASCs and intracellular lipid acumulation (oil red O staining)

**Fig. 2:**
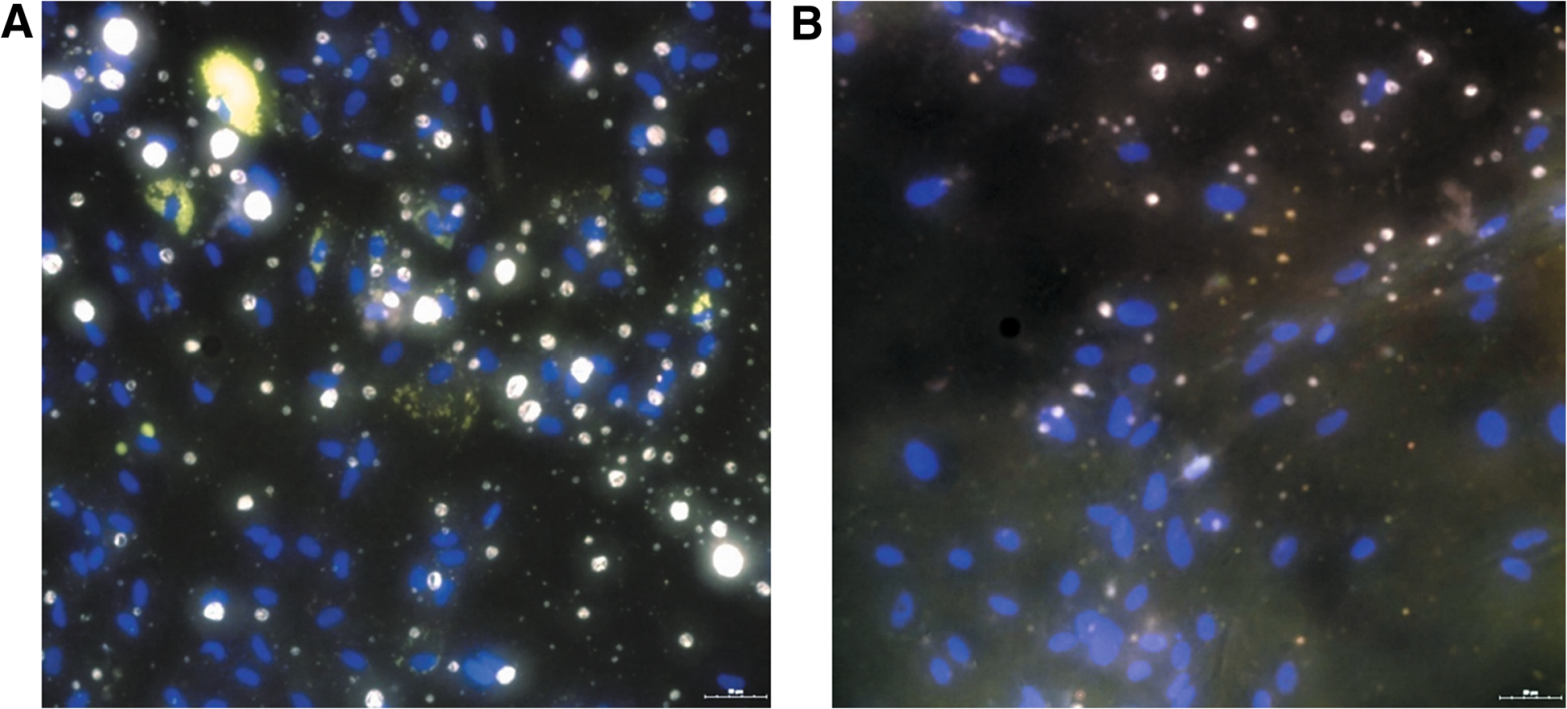
Immunostaining of adipogenic differentiated adipose-derived stem/stromal cells (ASCs) from NW (**a**) and obese (**b**) patients. Merge image of PPARγ (green), perilipin (yellow) and DAPI (blue)

Adipogenic differentiation protocol was monitored in cell cultures using optic microscopy and lipid accumulation was observed from day 10 ± 3 in NW samples and from day 12 ± 4 in OB. We observed fewer perilipin-positive (viable adipocytes) cells in OB saples as compared to NW samples (Fig. 2).

**Fig. 3:**
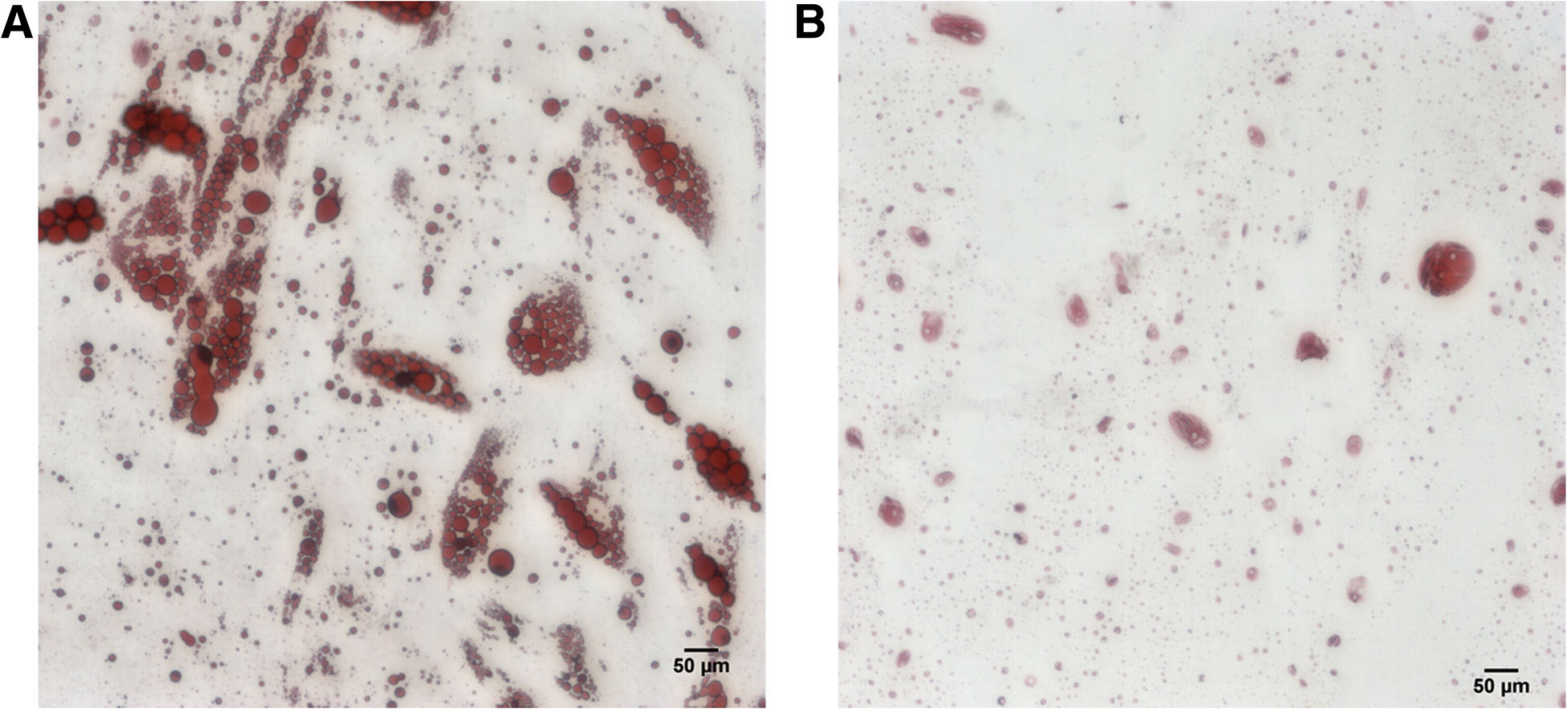
Specific lipid stain with ORO brightfield optic microscopy. **a**. Adipocytes differentiated from NW ASCs. **b**. Adipocytes differentiated from OB ASCs

Specific lipid stain was evaluated using ORO and the lipid accumulation obtained for adipocytes differentiated from NW (Fig. 3). The lipid droplets accumulated were fewer and smaller in the OB samples. ORO absorbance at 492 nm by spectrophotometry showed a significant increase (*p* < 0.001) in lipid deposition in ASCs derived from obese patients as compared to normal weight patients (Table 1).

### Correlation of lipid accumulation with metabolic parameters

Significant negative correlations were found between the lipid accumulation (OD_492_) and plasma triglycerides (*r* = − 0.42, *p* < 0.01), insulin (*r* = − 0.52, *p* < 0.001), HOMA-IR (*r* = − 0.49, *p* < 0.01), adiponectin (*r* = 0.32, *p* < 0.05), and leptin:adiponectin ratio (*r* = − 0.36, *p* < 0.05) (Table 2).

**Table 2:** Kendall’s Tau-b correlation coefficient of adipocyte lipid accumulation with anthropometric, metabolic and hormonal parameters

Co-variables	Adipocyte lipid accumulation	
	r	*P*-value
Age	0.01	0.95
BMI	−0.22	0.18
Total cholesterol (TC)	−0.04	0.82
LDL-cholesterol (LDL-C)	0.12	0.47
HDL-cholesterol (HDL-C)	0.08	0.65
TC/HDL	0.02	0.92
Triglycerides (TG)^*^	−0.42	0.01
Fasting plasma glucose (FPG)	−0.26	0.12
Insulin^*^	−0.52	0.001
C-peptide	−0.31	0.06
HOMA-IR^*^	−0.49	0.003
Morning cortisol	−0.1	0.53
Leptin	−0.22	0.16
Adiponectin^*^	0.32	0.05
Leptin:Adiponectin Ratio^*^	−0.36	0.03

r, Kendall’s Tau-b correlation coefficient. ^*^Significant correlation (*p* < 0.05)

## Discussion

The altered lipid accumulation capacity of subcutaneous preadipocytes in obese patients is currently being evaluated as a precocious marker for insulin resistance as it translates the incapacity of the mesenchymal cell line progenitors to differentiate mature, functional adipocytes with optimal lipid storage capacity. Several studies found that lipid accumulation evaluates the expansion capacity of the pre-adipogenic mesenchymal cell line and is associated with a poor metabolic profile for obese patients [24, 29–31].

The subcutaneous adipose tissue represents 90% of total fat mass, it has potential to greatly affect systemic insulin resistance via adipokine secretion, that plays an important role in glucose uptake impairment, as chemerin was reported to be associated positively with BMI and the markers of inflammation and metabolic syndrome in humans [22]. Adiponectin expression has been demonstrated to accelerate 3 T3-L1 proliferation and also lipid accumulation evaluated by Oil red O staining was found to be 4-fold greater in adipogenic differentiated pre-adipocytes that overexpressed adiponectin [32]. Also, in animal studies on mice the same results were found [33]. These results support the role of adiponectin as a key autocrine/paracrine factor that could play an essential role in the regulation of adipocyte metabolism and adipose tissue mass.

We report a decreased lipid accumulation capacity for adipogenic differentiated ASCs of obese female patients versus those from normal weight controls, and significant correlations between the adipocyte fat accumulation potential and insulin, adiponectin, leptin:adiponectin ratio (LAR).

In our study we did not find different levels of morning cortisol for obese patients and normoponderal controls. Also, there was no statistically significant correlation with lipid accumulation, showing that morning cortisol is not a valuable predictor for adipogenesis dysfunctions in obese patients. Literature data are inconsistent in this matter, some data suggest that glucocorticoids (GC) increase the lipid turnover in adipose tissue [34], but despite the major impact of GC on adipogenesis, normal or low circulating cortisol values are found in obese patients studies [35].

The fact that obesity alters the adipogenic differentiation capability of ASCs from subcutaneous adipose depots is supported by the data from other research groups [27, 36, 37]. De Girolamo et al. evaluated the ASCs adipogenic potential in obese versus non-obese controls and they also found a reduced proliferative rate for obese ASCs [31]. Alteration of the pre-adipocytes lineage in obese bariatric patients was also demonstrated by Perez et al. [37] that showed an altered lipid accumulation in obese-ASCs derived adipocytes as compared to adipocytes lineage coming from normal weight humans or mice. In another study, Perez et al. reported a significantly enhanced apoptosis and a reduced proliferative capacity of ASCs isolated from obese subjects; the impaired adipogenesis was correlated with the in vivo environmental obesity-related altered mitochondrial biogenesis, increased reactive oxygen species production and increased extracellular acidification [38].

We demonstrated that plasma insulin is correlated with decreased adipocyte ability to accumulate lipids. Previous research data published by Weyer et al., also show that the pre-adipocytes differentiation potential is correlated with insulin resistance in humans, in both obese and normal weight individuals [36]. Reported data from in vitro studies confirmed the decrease in the adipogenic differentiation ability of the ASCs from obese subjects and have described the mechanisms of obesity induced adipose-tissue remodeling, that include a disproportionate synthesis of extracellular matrix components [39] but also a decreasing number of adipocytes [40]. Thus, the variations in adipose cellularity that occur during the development of insulin resistance seem to determine a decrease in the clonogenic and proliferative potential of ASCs. Their role in unhealthy adipose tissue expansion associated with metabolic syndrome is an important predictor for obesity associated co-morbidities.

The novel finding in our study was the negative correlation between adipogenic potential (assessed by lipid accumulation in mature adipocytes) with triglycerides and LAR as key markers of impaired metabolic profile. These results point to abnormal adipogenesis as a link between obesity and its clinical complications.

Overall strengths of our study include direct measurement of the lipid accumulation in differentiated ASCs and the assessment of the relation between impaired adipogenesis with plasma metabolic and adipokine parameters in females with increased adiposity. A limitation of our study is the small size of the study and control groups and the relatively young age of the patients.

## Conclusions

Our study demonstrates that in severely obese female patients, the ASCs from subcutaneous adipose tissue have a decreased potential for adipogenesis as compared with normal weight controls. The abnormal lipid accumulation in the mature adipocyte derived from obese ASCs could possible predict the further metabolic changes and influence the selection of patients for bariatric surgery.

## Data Availability

The datasets used and/or analyzed during the current study available from the corresponding author on reasonable request.

## Abbreviations

ASCs: Adipose derived stem cells
HOMA-IR: Homeostatic Model Assessment for Insulin Resistance
LSG: Laparoscopic Sleeve Gastrectomy
MSCS: Mesenchymal stem cells
PPARγ: Peroxisome proliferator-activated receptor gamma
SVF: Stromal vascular fraction
